# Modulation of PICALM Levels Perturbs Cellular Cholesterol Homeostasis

**DOI:** 10.1371/journal.pone.0129776

**Published:** 2015-06-15

**Authors:** Jacob L. Mercer, Joseph P. Argus, Donna M. Crabtree, Melissa M. Keenan, Moses Q. Wilks, Jen-Tsan Ashley Chi, Steven J. Bensinger, Catherine P. Lavau, Daniel S. Wechsler

**Affiliations:** 1 Department of Pharmacology & Cancer Biology, Duke University, Durham, North Carolina, United States of America; 2 Department of Microbiology, Immunology and Molecular Genetics, Molecular and Medical Pharmacology, University of California Los Angeles, Los Angeles, California, United States of America; 3 Department of Pediatrics, Division of Pediatric Hematology-Oncology, Duke University, Durham, North Carolina, United States of America; 4 Department of Molecular Genetics and Microbiology, Duke University, Durham, North Carolina, United States of America; 5 Center for Genomic and Computational Biology, Duke University, Durham, North Carolina, United States of America; 6 Department of Radiology, Center for Advanced Medical Imaging Sciences, Massachusetts General Hospital, Boston, Massachusetts, United States of America; Instituto de Química, Universidade de São Paulo, BRAZIL

## Abstract

PICALM (Phosphatidyl Inositol Clathrin Assembly Lymphoid Myeloid protein) is a ubiquitously expressed protein that plays a role in clathrin-mediated endocytosis. PICALM also affects the internalization and trafficking of SNAREs and modulates macroautophagy. Chromosomal translocations that result in the fusion of PICALM to heterologous proteins cause leukemias, and genome-wide association studies have linked PICALM Single Nucleotide Polymorphisms (SNPs) to Alzheimer’s disease. To obtain insight into the biological role of PICALM, we performed gene expression studies of *PICALM*-deficient and *PICALM*-expressing cells. Pathway analysis demonstrated that *PICALM* expression influences the expression of genes that encode proteins involved in cholesterol biosynthesis and lipoprotein uptake. Gas Chromatography-Mass Spectrometry (GC-MS) studies indicated that loss of PICALM increases cellular cholesterol pool size. Isotopic labeling studies revealed that loss of PICALM alters increased net scavenging of cholesterol. Flow cytometry analyses confirmed that internalization of the LDL receptor is enhanced in *PICALM*-deficient cells as a result of higher levels of LDLR expression. These findings suggest that PICALM is required for cellular cholesterol homeostasis and point to a novel mechanism by which PICALM alterations may contribute to disease.

## Introduction

PICALM (Phosphatidyl Inositol Clathrin Assembly Lymphoid Myeloid protein) is a ubiquitously expressed accessory adaptor protein that functions in Clathrin-Mediated Endocytosis (CME) [1–3]. PICALM’s role in CME is mediated by its N-terminal ANTH domain that allows it to interact with Phosphatidylinositol 4,5-bisphosphate (PIP_2_) and associate with the plasma membrane [4]. PICALM also binds to the clathrin heavy chain through a poorly defined clathrin binding region in its C terminal region [5]. PICALM regulates the internalization and localization of specific cell surface proteins, such as the transferrin receptor (TfR) and the epidermal growth factor receptor (EGFR). Loss of PICALM results in perturbed internalization of TfR and EGFR, while PICALM overexpression has a dominant negative effect on the internalization of these receptors [2, 3, 5–7]. In addition, PICALM plays a key role in regulating vesicle size, since PICALM deficiency results in enlarged and elongated vesicular formation [2, 8, 9].

PICALM also regulates the internalization and localization of specific soluble NSF attachment protein receptors (SNAREs), including VAMP2, VAMP3, and VAMP8 [8, 10–13]. These SNAREs play important roles in vesicular trafficking, including the endosomal-lysosomal system and macroautophagy [8, 10–13]. In the absence of PICALM, specific SNAREs are mislocalized and retained at the plasma membrane [12, 13]. We recently showed that PICALM modulates macroautophagy as a result of its ability to regulate SNARE protein localization [13]. The effect of PICALM on the cellular localization of SNAREs may impact other cellular processes involving trafficking. Indeed, a recent study indicated a role for PICALM perturbation in endosomal-lysosomal maturation affecting the intracellular trafficking of the cKIT receptor [14].

In addition to its role in endocytosis and SNARE localization in the cytoplasm, a fraction of PICALM can also be found in the nucleus. PICALM contains a Nuclear Export Signal (NES) that allows it to shuttle between the nucleus and the cytoplasm. The role of PICALM within the nucleus is presently not known, although it can activate gene transcription in reporter assays [15].

PICALM alterations have been associated with several human diseases. The *PICALM* gene was initially identified as a site of chromosomal translocation in a cell line derived from a lymphoma patient [16]. In the U937 cell line, *PICALM* is fused to *AF10* as a result of a t(10;11) chromosomal translocation that has since been described in a variety of hematologic malignancies, including acute lymphoid and myeloid leukemias [17, 18]. We recently reported that CALM contributes to the leukemogenic properties of CALM-AF10 as a result of its NES motif [19, 20].

Several Genome Wide Association Studies (GWAS) have reported that specific single nucleotide polymorphisms (SNPs) within *PICALM* are associated with the development of late onset Alzheimer’s Disease (LOAD) [21–24]. However, the mechanisms by which PICALM contributes to the development of LOAD are unknown. Recent studies suggest that PICALM may be involved in the production of toxic amyloid beta plaque formation [25]. Moreover, PICALM is involved in macroautophagy and can affect Tau accumulation, both of which may play essential roles in the development of LOAD [13].

To gain insight into the biology of PICALM, we performed microarray studies comparing gene expression in *Picalm* knockout and *PICALM*-rescued cells. Pathway analysis suggested that PICALM perturbation results in altered expression of genes in the cholesterol biosynthesis pathway. Using quantitative PCR, we confirmed that PICALM overexpression and PICALM loss results in elevated cholesterol biosynthesis pathway gene expression in multiple cell types. Isotopic labeling studies demonstrated that reduction of PICALM levels results in both increased cellular cholesterol levels and net scavenging rate. Collectively, these studies suggest that PICALM plays a role in cellular cholesterol metabolism, highlighting a novel mechanism by which alterations of PICALM might contribute to disease.

## Materials and Methods

### Ethics Statement

All *in vivo* and euthanasia procedures in this study were carried out in strict accordance with the recommendations in the Guide for the Care and Use of Laboratory Animals of the National Institutes of Health. Adult mice were euthanized by CO_2_ exposure and fetuses were euthanized by decapitation. The animal studies described here have been approved by the Duke University Institutional Animal Care & Use Committee (IACUC) (Protocol# A021-13-01). All efforts were made to minimize animal suffering.

### Animal Welfare

The animal studies described here have been approved by the Duke University Institutional Animal Care & Use Committee (IACUC) (Protocol# A021-13-01). Adult mice were euthanized by CO_2_ exposure and fetuses were euthanized by decapitation. None of the experiments performed involved animal suffering.

### Cell Culture

HEK293 cells (ATCC, catalog # CRL-1573), MEFs [5] and retroviral packaging Plat-E cells (a gift from T. Kitamura) [26] were maintained in DMEM (Gibco, Life Technologies, Grand Island, NY, USA) supplemented with 10% fetal bovine serum (Gibco, Life Technologies), 1% penicillin/streptomycin (Gibco, Life Technologies,). For lipid withdrawal experiments, HEK293 cells were grown with 10% lipid deficient fetal calf serum (Sigma). CAD neuronal cells (a gift from Dona Chikarashi (Duke University) in March 2012) [27, 28] were cultured in DMEM/F12 (Gibco, Life Technologies) supplemented with 8% fetal bovine serum (FBS), penicillin and streptomycin (Invitrogen, Carlsbad, CA, USA). HEK293 and Plat-E cells were transfected by calcium chloride transfection [29]. HEK293 cells transfected with pRSMX vectors were selected with puromycin (Sigma-Aldrich, St. Louis, MO, USA). MEFs and CAD cells were infected by co-culture with filtered (0.45 uM filter) Plat-E supernatant in the presence of polybrene (Sigma-Aldrich) (2 μg/ml). Hematopoietic cell lines were generated by immortalizing fetal liver cells harvested from E14 *fit1 Picalm* ^*fit1-5R*^ mouse embryos [5]. *fit1 Picalm* ^*fit1-5R*^ mice have a mutation that codes for a non functional Picalm protein missing 82% of its amino acid sequence ([30]), herein referred to as *Picalm* ^-/-^. Fetal liver cells were retrovirally transduced with MLL-ENL using the MSCV-IRES-eGFP vector [31] and serially replated in methylcellulose culture to select immortalized hematopoietic progenitors as previously described [32]. Cells were maintained in liquid culture in RPMI medium supplemented with 10% FBS, glutamine, penicillin and streptomycin (Invitrogen) with recombinant murine interleukin-3 and stem cell factor (5 ng/ml and 50 ng/ml, respectively, Peprotech, Rocky Hill, NJ, USA). The doubling times for cells used within the study were calculated using Eq 1. c(t) = cell number at time t, t = time, c0 = cell number at time 0, k = exponential constant.

c(t)=c0*e^kt Eq 1

### DNA Constructs and Vectors

PICALM shRNA were expressed using the pRSMX retroviral vector (kindly provided by Louis Staudt (NIH)) [33]. An shRNA directed against luciferase (5’ GTGGATTTCGAGTCGTCTTTAAT-3**’**) was used as a negative control. Several shRNAs were used to knock down PICALM in human or murine cells: *Picalm* shRNA4 (5**’**-GCCTTAATGTTGACTTTGAAT-3**’**), *Picalm* shRNA10 (5”- GAAATGGAACCACTAAGAA-3’), *Picalm* shRNA15 (5’-GAGCACAGATACAGTTTAT-3’). All shRNAs were cloned into *HindIII and BglII sites*. shRNA4 targets human and mouse PICALM. shRNA10 targets human and mouse PICALM, however, results in no knockown of human PICALM in HEK293 cells. shRNA15 targets human and mouse PICALM but results in a very modest knockown of PICALM in mouse cells. *Picalm* knockout fetal liver hematopoietic cells were rescued by retroviral transduction of human PICALM cDNA using the MSCVneo retroviral vector [34]. Control cells were infected with the MSCVneo empty vector. Transduced cells were selected in G418 (Geneticin; Gibco, Life Technologies). Transfections into HEK293 cells were performed using calcium chloride transfection [29]. PICALM overexpression experiments in HEK293 cells were performed using human *PICALM* cDNA [5] subcloned into the bicistronic MSCV-IRES-eGFP (MIE) retroviral vector. The HEK293 control cells were transfected with the MSCV-IRES-eGFP (MIE) empty vector.

### Antibodies and Reagents

The following reagents were used: rabbit anti-PICALM antibody (HPA019061, Sigma-Aldrich, 1:1000), mouse anti-actin antibody (AC-40, Sigma-Aldrich, 1:1000), rabbit anti-actin antibody (A2066, Sigma-Aldrich, 1:1000), rabbit anti-SREBP2 antibody (ab28482, 1:1000, Abcam, Cambridge, MA, USA,), IRDye680-conjugated donkey anti-rabbit antibody (926–68073, 1:5000, LI-COR, Biosciences, Lincoln, NB, USA), PE-conjugated mouse anti-human LDLR antibody (FAB2148P, 1:20, R&D Systems, Minneapolis, MN, USA), PE-conjugated mouse IgG Isotype antibody (IC002P, 1:20, R&D Systems).

### Western Blot Analysis

Cells were lysed in Laemmli buffer and lysates were resolved by SDS–PAGE electrophoresis (using 7.5% acrylamide gels) and transferred to polyvinylidene difluoride membranes (IPFL00010, Millipore, Billerica, MA, USA). Membranes were incubated with the appropriate antibody overnight in Odyssey Blocking Buffer (Li-Cor) with 0.1% Tween-20. Membranes were washed in TBS with 0.1% Tween-20. Secondary antibody incubation was performed in Odyssey Blocking Buffer with 0.1% Tween-20 and 0.02% SDS for 30 min. Membranes were washed and proteins were quantitated using an Odyssey Infrared fluorescence imaging system (Li-Cor). All uncropped western blots are included as **S5 Fig.**

### Microarray

Total RNA was isolated using Qiagen RNeasy Micro kit (Qiagen, Valencia, CA) according to the manufacturer’s protocol and analyzed by Mouse Genome 430 2.0 Array (Affymetrix, Santa Clara, CA, USA). Array data were then normalized by RMA for further analysis. To identify differentially expressed genes, we applied the SAM 4.01 Excel Add-In that provides the estimate of False Discovery Rate (FDR) for multiple testing. Using an FDR threshold of 12.3%, we identified 343 probe sets, whose expression values were extracted, mean-centered [35] and clustered by Cluster 3.0 and displayed by Treeview v1.6 as done [36] These selected genes were then deposited into GATHER (gather.genome.duke.edu) [37] to determine the enrichment for the Gene Ontology (GO) and KEGG (KEGG). Specifically, KEGG pathway analysis was used in this study. All array analysis was conducted using log2 scale. The data discussed in this publication have been deposited in NCBI’s Gene Expression Omnibus and are accessible through GEO Series accession number GSE64855.

### Quantitative RT-PCR Analysis

Total RNA was isolated from MEFs using RNeasy Mini Kit (Qiagen). RNA was reverse transcribed using iScript cDNA Synthesis Kit (Bio-Rad, Hercules, CA, USA) according to the manufacturer’s protocol. Real time quantitative PCR was performed using iQ Sybr-Green Mix (Bio-Rad) according to the manufacturer’s protocol. Amplification data were collected using the iQ5 Optical System (Bio-Rad). The expression levels of cholesterol biosynthesis genes were normalized to beta 2-microglobulin (β2M) and then to empty vector or shRNA luciferase control cells by the ΔΔCT method. Primer pair sequences are provided in **S1 Table** and **S2 Table**.

### Surface Expression and Internalization Assays

HEK293 cells growing exponentially were trypsinized with 0.05% Trypsin/EDTA (Invitrogen) until approximately 30% of cells lifted off the plate. Trypsinization was stopped with serum-containing ice cold media. Cells were washed with cold PBS and resuspended in HFN (Hanks Balanced Salt Solution, FBS (2%), Sodium Azide (NaN3 0.02%) containing rat IgG (1 μg/ml) for 20 min. Cells were then stained with anti-human LDLR-PE or mouse PE-conjugated IgG Isotype control for 30 minutes on ice. Cells were washed twice with cold PBS and then directly analyzed by flow cytometry (C6, Accuri, Ann Arbor, MI, USA) to measure LDL receptor surface expression by quantification of mean fluorescence intensity. For internalization assays, cells stained with antibodies and rinsed in PBS were divided into multiple tubes and incubated at 25°C for indicated time points to induce internalization. Cells were incubated at 25°C rather than 37°C to slow down LDL receptor internalization and improve accuracy of measurements. Ice-cold acid wash buffer (0.5 M NaCl/0.2 M acetic acid) was added to halt receptor internalization and strip residual LDL receptor from the cell surface. Cells were washed twice with cold PBS and flow cytometry was used to measure the mean fluorescence intensity emitted by internalized LDL receptor molecules. The rate of LDL receptor internalization was calculated by dividing the LDL receptor internalized at each time point by the initial LDL receptor surface expression.

### Lipid Quantitation and ^13^C Tracer Analysis

HEK293 cells were cultured in glucose-free DMEM (Gibco, Life Technologies) supplemented with 10% FBS, 1% pen/strep, and 25 mM glucose. Unlabeled cells were cultured in natural glucose (Sigma-Aldrich), while labeled cells were cultured in a 1:1 molar ratio of natural to U-^13^C_6_-glucose (110187-42-3, Cambridge Isotopes, Andover, MA, USA). To reach steady state, cells were cultured in natural or label-containing medium for 6 d. The medium was changed every 12 hours. Cells were grown at the same cell density and were maintained in a subconfluent state by splitting every 48 hours. After 6 d (144 h) in natural or label-containing medium, cells were collected, counted, washed, and frozen at -80°C. To obtain growth curves, HEK293 shRNALuc and shRNA4 cells were grown in quadruplicate in the natural and label-containing medium after splitting the cells on d4. Cells were counted 24 and 48 h after the initial plating in order to measure cellular growth rates. Doubling time was determined by fitting cell counts at 24 h (120 h–d5) and 48 h (144 h–d6) to an exponential curve using Excel (Microsoft).

Calibration curves were made using the GLC-96 fatty acid methyl ester (FAME) mix (Nu-Chek Prep), methyl cis-vaccenate (Nu-Chek Prep), and cholesterol (Sigma). 4 μg of trinonadecanoin (Nu-Chek Prep) and stigmastanol (Sigma-Aldrich) were added in 400 μL of toluene to each sample as internal standards. 3 mL of methanol and 600 μL of 8% (w/v) HCl in methanol were added to each sample before incubating at 45°C for 16 h. 2 mL of 0.04 M aqueous NaCl was added to the reaction mix and FAMEs and free sterols were extracted twice using 2 mL of n-hexane. The combined organic phases were dried under vacuum (EZ-2 Elite) and dissolved in 300 μL n-hexane. One hundred μL of this solution was used directly for FAMEs analysis via GC/MS. 150 μL of this solution was dried again under vacuum and dissolved in 25 μL of 1:1 (v/v) pyridine and *N*,*O*-bis(trimethylsilyl) trifluoroacetamide with trimethylchlorosilane 99:1 (Sigma-Aldrich). The resulting trimethylsilyl derivatives were directly analyzed via GC/MS.

Data was collected on an Agilent 5975C MSD (Agilent Technologies, Santa Clara, CA, USA) connected to an Agilent 7890A Gas Chromatograph (Agilent). FAMEs were analyzed on the Agilent DB-WAX column (122–7032). Trimethylsilyl ether sterols were analyzed on a ZB-MR-1 column (7HG-G016-11, Phenomenex, Torrance, CA, USA). GC/MS settings and oven programs are available upon request. Ions monitored for each analyte are provided in **S3 Table**. Area under the curve (AUC) quantitation was conducted on ChemStation software (Agilent). Absolute quantitation of fatty acids and cholesterol was achieved by first normalizing the analyte AUC (sum of all collected isotopologues) to the internal standard AUC (M+0 only). This ratio was fit to the appropriate calibration curve and then normalized to cell number. The relative contributions of synthesis and scavenging were determined by fitting the isotopologue distributions at steady state (>5 divisions in label media) by modeling the isotopologue distribution as described in [38, 39]. Note that for 16:1(n-7), the “scavenged” population includes scavenged 16:0 desaturated by stearoyl-CoA desaturase 1 (SCD1) inside the cell. Net accumulation rates (R) were calculated using pool size (p), doubling time (d), and percent contributions (c) using Eq 1. “Net rate” for a given metabolic parameter (e.g. synthesis) is defined as the gross rate minus the rate of loss due to modification/breakdown/export.

$$R=\ln\left(2\right)\frac{cp}{d}$$ Eq 2

### Statistical Analysis

Standard, two-tailed Student’s t-Test analysis was used for all statistical analysis. Values represent means ± SEM from at least three independent experiments. p values less than 0.05 were considered statistically significant.

## Results

### *PICALM* Affects Cholesterol Biosynthesis Gene Expression

To better understand PICALM’s basic biological role, we established cell lines from *Picalm* ^*-/-*^ mice [5, 30] to conduct microarray analyses. Immortalized fetal hematopoietic cells from E14 *Picalm* ^-/-^ embryos were retrovirally transduced to rescue *PICALM* expression. Control *Picalm* ^-/-^ cells were transduced with an empty MSCVneo retroviral vector. Four biological replicates of *Picalm* ^-/-^ cell lines, transduced with either *PICALM* or the empty vector, were used to perform the microarray.

**Fig 1:**
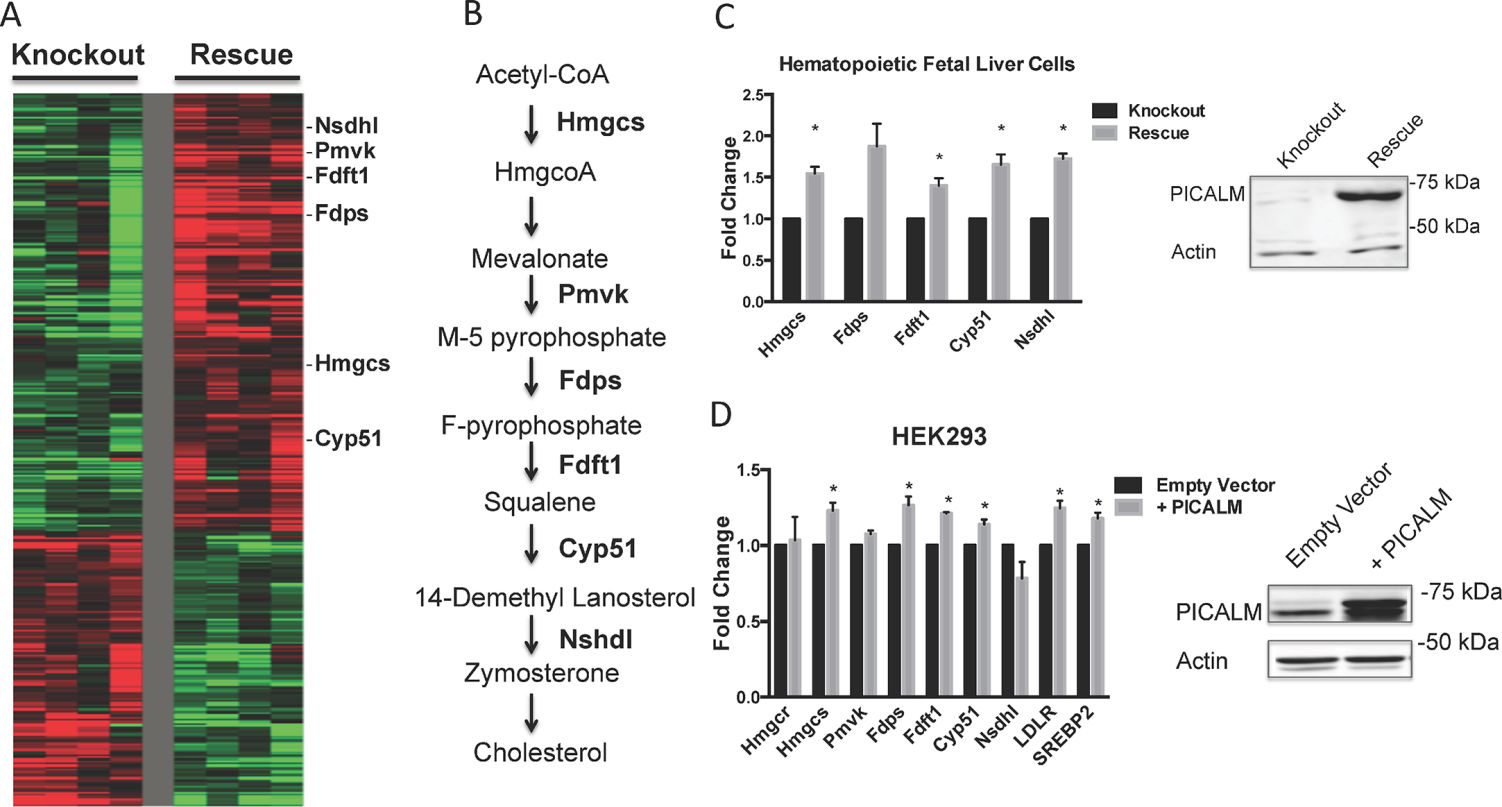
Microarray analysis identifies altered cholesterol biosynthesis gene expression in *Picalm*-knockout and *PICALM*-rescued cells. **(A)** Heatmap comparing microarray gene expression profile of four *Picalm*-knockout (left panels) and four *PICALM*-rescued (right panels) murine hematopoietic cell lines. Red indicates genes that are upregulated, green indicates those that are downregulated. Genes of the cholesterol pathway are indicated to the right of the heatmap. **(B)** Simplified diagram of cholesterol biosynthesis pathway indicating enzymes that are overexpressed in *PICALM*-rescued cells by the microarray study. **(C)** Validation of microarray results by qPCR in hematopoietic fetal liver cells (upper panel). Expression is normalized to *Picalm*-knockout cells. Data represent the mean of 3 independent experiments. *p<0.05. Immunoblot (lower panel) shows PICALM and actin protein levels in representative *Picalm*-knockout and *PICALM*-rescued hematopoietic cells. Doubling times of fetal liver cells (14 hours) and HEK293 cells (11 hours) were calculated using Eq 1 in Materials and Methods. **(D)** Cholesterol biosynthesis gene expression was measured in HEK293 cells transiently transfected with a control retrovirus (Empty Vector) or PICALM expressing retroviral vector (+PICALM). Data represent the mean of 3 independent experiments. Results are shown normalized to control cells. *p<0.05. Immunoblot in right panel shows levels of PICALM and actin proteins.

We used the GATHER (Gene Ontology Tool to Help Explain Relationships) gene ontology tool to identify common pathways altered in *Picalm* knockout versus *PICALM*-rescued cell lines [37]. This analysis revealed that nine GO terms involving lipid and cholesterol synthesis (including GO:0006695, 0044255, 0016126, 0008610) were among the ten most significantly altered pathways when comparing *Picalm* ^-/-^ and *PICALM*-rescued cells (**Table 1**). Expression levels of multiple genes in the cholesterol biosynthesis pathway were elevated in *PICALM*-rescued compared with *Picalm* ^-/-^ cells (**Fig 1A and 1B**). These included *Hmgcs* (1.2 fold), *Fdps* (1.3 fold), *Fdft1* (1.3 fold), *Cyp51* (1.2 fold) and *Nsdhl* (1.2 fold). Several genes involved in basic cellular lipid metabolism were also increased in the presence of *PICALM*, including *Dgat2* (1.5 fold), *Mogat2* (2.4 fold), *Slc27a4* (1.3 fold) and *Pip5k1b* (1.5 fold). Although some of the inductions were modest, the concordant changes in numerous genes of the cholesterol biosynthesis pathway strongly suggest that PICALM expression levels play a role in cholesterol metabolism. Validation of the induction of cholesterol biosynthesis gene expression in *PICALM*-rescued cells was performed by quantitative real time PCR (qPCR) (**Fig 1C**) and confirmed elevations in cholesterol pathway gene expression. The top 40 up-regulated and down-regulated genes in the microarray analysis are shown in **S4 and S5 Tables**, respectively.

**Table 1:** Top Ten Biological Pathways Elevated in the Presence of PICALM*.

Number	GO term	neg ln (p value)	Genes
**1**	GO:0006695 [8]: cholesterol biosynthesis	10.41	**Cyp51, Fdft1, Hmgcs1, Nsdhl, Pmvk**
**2**	GO:0044255 [5]: cellular lipid metabolism	10.41	Alox5ap, Cdipt, **Cyp51**, Dgat2, **Fdft1**, **Fdps**, Galc, **Hmgcs1**, Hsd11b1, Mogat2, Nsdhl, Pip5k1b, **Pmvk**, Slc27a4
**3**	GO:0016126 [7]: sterol biosynthesis	10.41	**Cyp51, Fdft1, Hmgcs1, Nsdhl, Pmvk**
**4**	GO:0008610 [6]: lipid biosynthesis	10.41	Alox5ap, Cdipt, **Cyp51**, Dgat2, **Fdft1, Fdps, Hmgcs1**, Mogat2**, Nsdhl, Pmvk**
**5**	GO:0006629 [5]: lipid metabolism	10.41	Alox5ap, Cdipt, **Cyp51**, Dgat2**, Fdft1, Fdps**, Galc, **Hmgcs1**, Hsd11b1, Mogat2, Nsdhl, Pip5k1b, Plcb2, **Pmvk**, Slc27a4
**6**	GO:0008203 [7]: cholesterol metabolism	9.16	**Cyp51, Fdft1, Hmgcs1, Nsdhl, Pmvk**
**7**	GO:0016125 [6]: sterol metabolism	8.8	**Cyp51, Fdft1, Hmgcs1, Nsdhl, Pmvk**
**8**	GO:0006066 [5]: alcohol metabolism	8.62	**Cyp51, Fdft1,** Gpd2, Hk1**, Hmgcs1, Nsdhl**, Pmm1, **Pmvk**
**9**	GO:0006694 [7]: steroid biosynthesis	8.16	**Cyp51, Fdft1, Hmgcs1, Nsdhl, Pmvk**
**10**	GO:0007186 [6]: G-protein coupled receptor protein signaling pathway	7.85	Arrb2, Ccr5, Ltb4r1, Mrgpra2

* Genes selected for further study are indicated in **boldface.**

The comparison of *Picalm* ^-/-^ with *PICALM*-rescued cells demonstrates that *PICALM* gene dosage affects the expression of cholesterol biosynthesis genes. However, one caveat of this experimental approach is that the level of *PICALM* expression in rescued cells may exceed physiological amounts; this could potentially squelch PICALM binding partners and have a dominant negative effect that exceeds the effect resulting from mere loss of PICALM [3, 5]. To examine this possibility, we compared wild type HEK293 cell, to cells transiently overexpressing PICALM. We performed qPCR to compare the expression of cholesterol biosynthesis genes in the empty vector control cells to that in the PICALM overexpressing cells. The results show that PICALM overexpression induces an increase of cholesterol metabolism gene expression (**Fig 1D**) suggesting that PICALM overexpression perturbs cholesterol homeostasis. This is consistent with studies showing that PICALM overexpression results in a dominant negative effect on multiple processes, including clathrin mediated endocytosis ([3, 6], iron metabolism [5] and macroautophagy (our unpublished data).

**Fig 2:**
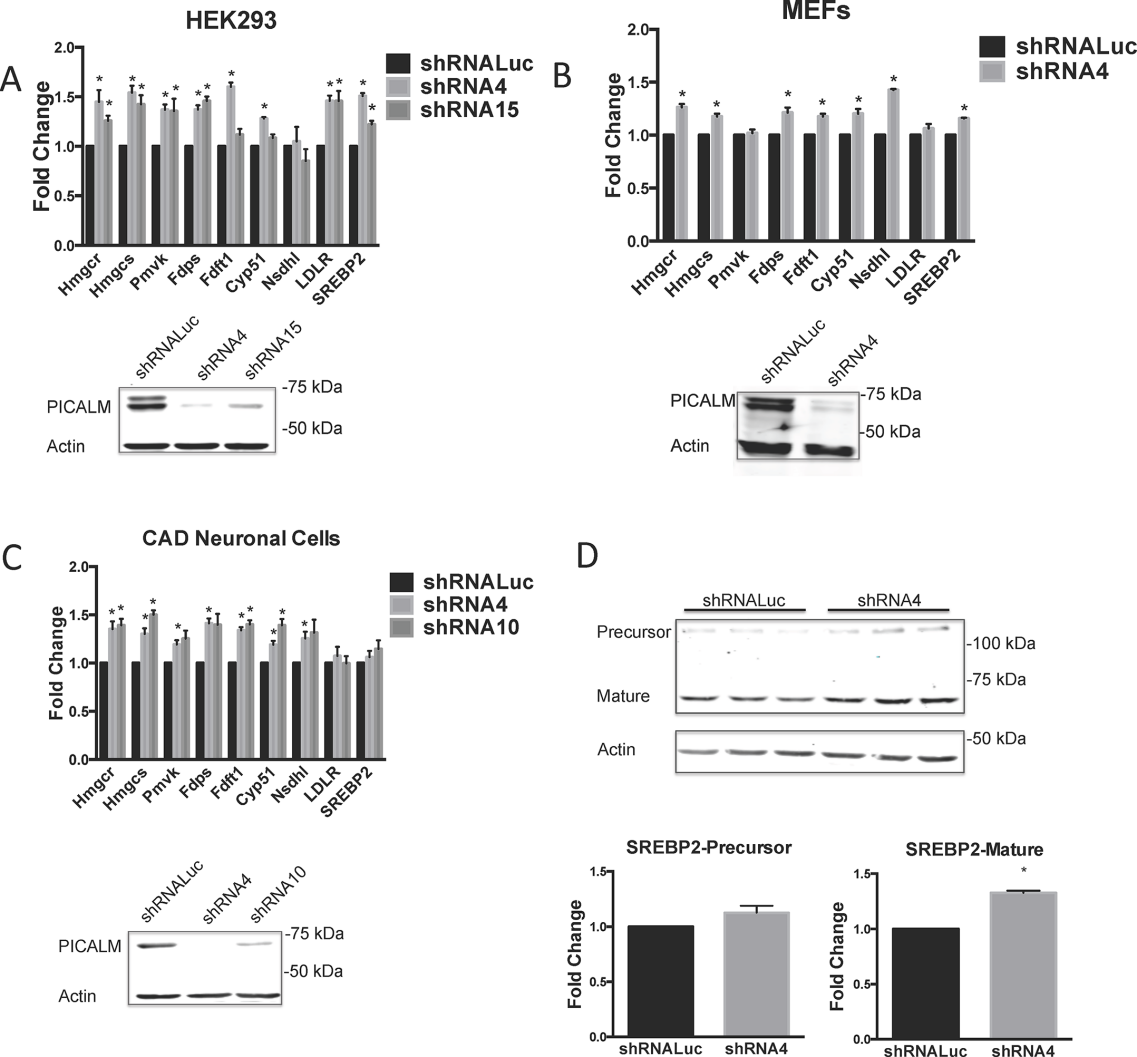
*PICALM* knockdown leads to increased cholesterol biosynthesis gene expression in multiple cell types. Cholesterol biosynthesis gene expression asessed by qPCR in cells expressing a *luciferase* control shRNA (shRNALuc) or various *PICALM* shRNAs (shRNA4, 10 or 15). Analysis was done in HEK293 cells **(A)**, MEFs **(B)** and CAD Neuronal cells **(C)**. Expression levels are normalized to those in shRNA control cells. Results shown are mean of three or more independent experiments. *p<0.05. Expression analysis of PICALM and actin in the various cell lines are shown by immunoblot in lower panels. Doubling times of cells were calculated using Eq 1 in Materials and Methods, HEK293, 11 hours, MEFs 14 hours, and CAD cells, 18 hours. **(D)** Levels of SREBP2 precursor (120 kDa) and mature form (70 kDa) measured by immunoblot in HEK293 cells expressing shRNA4 or shRNALuc control. Triplicate cell lysates were analyzed. Lower panel demonstrates quantitation of SREBP2 precursor and mature forms normalized to actin, with values shown relative to levels in shRNA control cells. *p<0.05.

To elucidate and further validate the role of PICALM in cholesterol homeostasis, we knocked down *PICALM* with shRNAs and compared cholesterol biosynthesis gene expression in cells expressing a control shRNA. Using two separate shRNAs to knockdown *PICALM* in HEK293 cells, we found a 20 to 50% increase in expression of cholesterol biosynthesis genes in *PICALM*-deficient cells (**Fig 2A**).

To extend this observation to additional cell lines, we knocked down *Picalm* in murine embryonic fibroblasts (MEFs). Similar to observations in HEK293 cells, we found that cholesterol biosynthesis gene expression increased by 10–50% in *Picalm*-deficient MEFs (**Fig 2B**). We also studied the effect of *Picalm* knockdown in the murine CAD (Cath-a-differentiated) neuronal cell line, a central nervous system catecholaminergic cell line established from a mouse brain tumor [27, 28]. Reduction of *Picalm* expression in CAD neuronal cells resulted in a 20–50% increase in cholesterol biosynthesis gene expression (**Fig 2C**).

We found that growing cells under lipid-deprived conditions (5 and 24 hours) increases the expression of cholesterol biosynthesis genes. In the absence of lipids, the levels of expression of these genes are not significantly different between *PICALM*-deficient and control HEK293 cells (**S1 Fig**). These results suggest that the *PICALM*-deficient cells display a lesser response to lipid starvation, possibly because they are already sensing lipid deprivation under normal growth conditions.

Finally, to further examine the effect of PICALM perturbation on cholesterol homeostasis, we studied its effect on expression of SREBP2, a key regulator of cholesterol metabolism gene expression [40]. SREBP2 is present in a precursor (120 kDa) inactive form, and a mature (65 kDa) active form that activates the transcription of cholesterol biosynthesis genes. To complement the gene expression analysis, we found that *PICALM* loss is associated with a modest elevation of the mature 65 kDa SREBP2 form in HEK293 cells (**Fig 2D**). Similar results were seen with shRNA15 (**S2A Fig)**. Of note, we have observed that changes in levels of SREBP2 mature/active form seen by immunoblotting are not striking; even upon growing HEK293 cells in lipid deficient conditions for 24 hours–conditions that would be expected to dramatically increase SREBP2 levels–we only see a 45% increase in the mature SREBP2 product (**S2B Fig**). The gene expression analysis along with a subtle elevation of SREBP2, indicate that cholesterol homeostasis is altered when PICALM expression is perturbed.

### PICALM Loss Enhances Cellular Cholesterol and LDL Receptor Internalization

**Fig 3:**
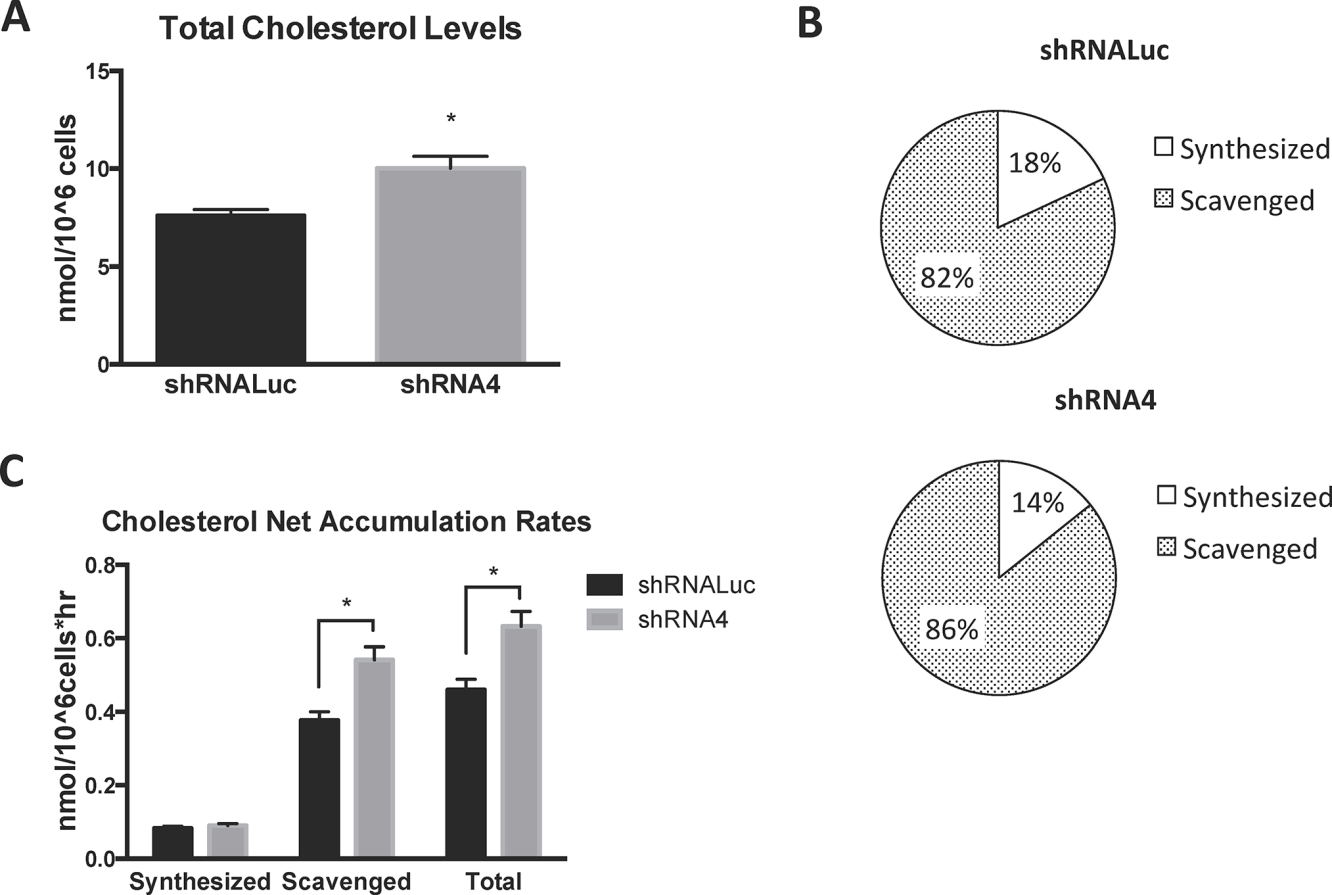
Total cholesterol levels, net scavenging, and net total accumulation are elevated in *PICALM* knockdown cells. **(A)** Total cellular cholesterol levels were measured in HEK293 cells expressing a control (luciferase) shRNA or *PICALM* shRNA (shRNA4). All results were normalized to total cell number. n = 4; *p<0.05. **(B)** The relative contribution of synthesis and scavenging to cellular cholesterol was calculated at steady state by modeling isotopologue distribution of cholesterol in indicated cells cultured in 1:1 molar ratio of natural to U-^13^C_6_-glucose (n = 4 in each experimental group). **(C)** Net rates of cholesterol synthesis, scavenging and total accumulation were calculated using cholesterol pool size, relative contribution of synthesis and scavenging to cholesterol pool, and doubling time of cells as described in Materials and Methods. n = 4 in each experimental group; *p<0.05.

Our studies demonstrate that loss of PICALM results in elevated expression of genes that are involved in both cholesterol biosynthesis and lipoprotein uptake. We reasoned that this gene signature might be the result of decreased cellular cholesterol content leading to a compensatory increase in cholesterol biosynthesis or increased cholesterol scavenging. To address this possibility, total cellular cholesterol was analyzed by Gas Chromatography-Mass Spectrometry (GC-MS). Unexpectedly, these studies revealed that knockdown of *PICALM* in HEK293 cells results in *increased* total cellular cholesterol levels (**Fig 3A**). We therefore performed ^13^C tracer analysis to determine if the relative contributions of synthesis and scavenging to the cellular cholesterol pool had changed. *PICALM* knockdown and control cells were cultured to steady state in complete media containing a 1:1 molar ratio of U-^13^C_6_ glucose and natural glucose [39] and total cellular cholesterol was extracted, derivatized and measured by GC-MS. The isotope distribution for cholesterol was assessed, and relative contribution of synthesis versus scavenging was determined by modeling the isotopologue distribution (see Methods for detailed protocol) [39]. We observed that PICALM knockdown cells had a higher percent abundance of unlabeled cholesterol, suggesting an increased relative reliance on scavenging compared to synthesis (**Fig 3B**). To better understand the relative importance of scavenging and synthesis, we calculated the net rates of accumulation (hereafter referred to as “net rates”) for synthesis and scavenging, which represent the gross rate minus losses due to modification, breakdown, or export (see Methods). *PICALM* knockdown did not appear to affect the net rate of cholesterol biosynthesis (**Fig 3C**). In contrast, we found that *PICALM* knockdown results in significantly elevated net rates of cholesterol scavenging and total cholesterol accumulation (**Fig 3C**). Several species of fatty acids also showed increased cellular pool sizes and increased net scavenging rates in the absence of PICALM (**S3 and S4 Figs**). Taken together, these results indicate that reduced PICALM expression is associated with increased cellular cholesterol content and a relative increase in the contribution of lipoprotein scavenging to cholesterol homeostasis.

**Fig 4:**
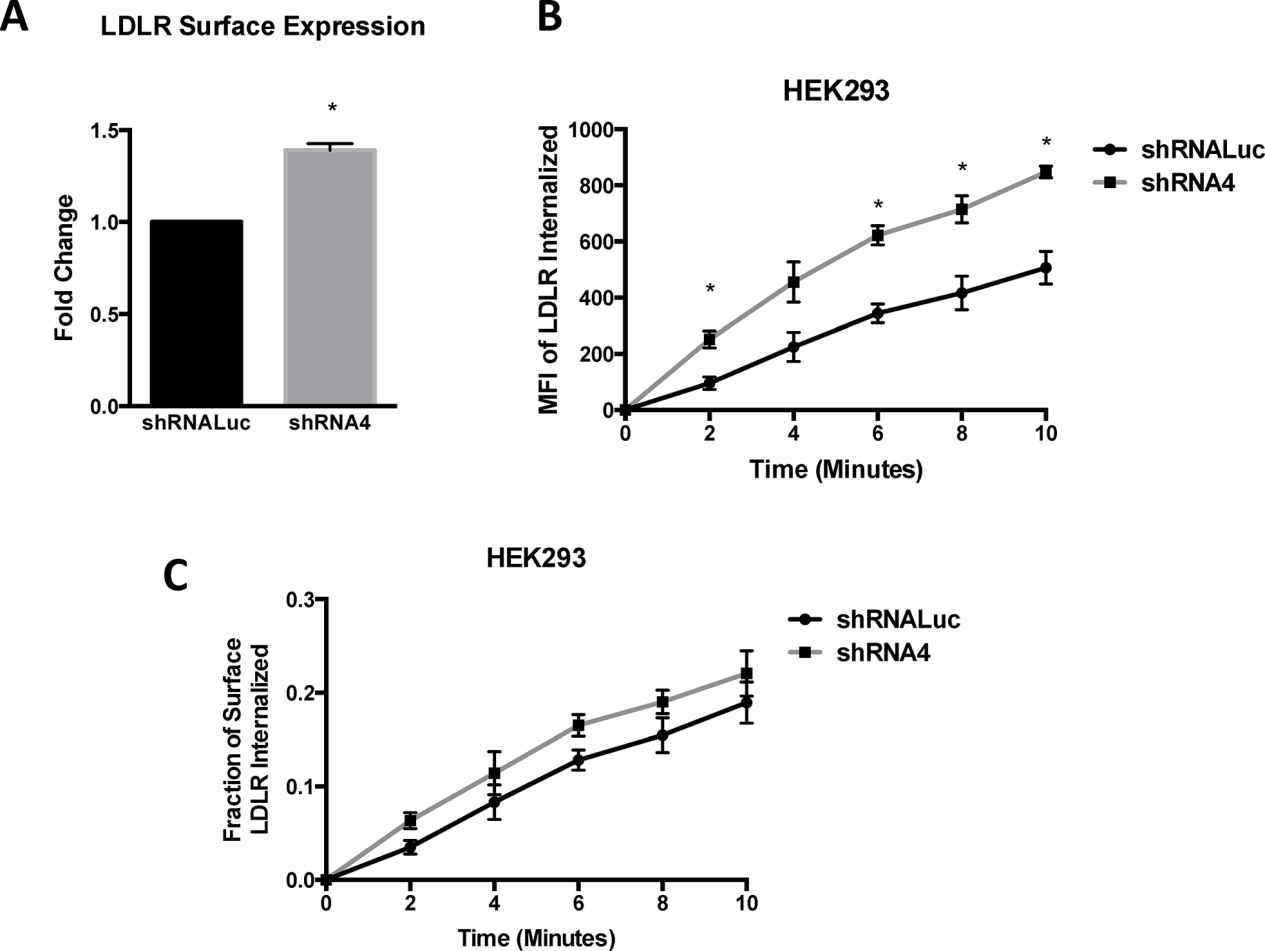
*PICALM* shRNA knockdown increases LDL receptor surface expression and internalization. **(A**) LDL receptor surface expression measured by flow cytometry in HEK293 cells expressing a *luciferase* shRNA (shRNALuc) control or *PICALM* shRNA (shRNA4). Expression levels are normalized to shRNALuc control cells. Results represent the mean of at least three independent experiments. *p<0.05. Endocytosis assays show the net amount of LDL receptor internalized **(B)** and the fraction of internalized LDL receptor relative to the initial amount of surface LDL receptor **(C)**, as a function of incubation time at 25°C in shRNALuc control or *PICALM* shRNA (shRNA4) cells. Each data point is the mean of three independent experiments. MFI = mean fluorescence intensity.

The LDL receptor is one of the principal routes by which cholesterol enters into the cell; its internalization is dependent on clathrin-mediated endocytosis (CME) [41–43]. Since PICALM plays a well-established role in CME, we sought to determine how reduced PICALM expression affects LDL receptor internalization. As shown in **Fig 4A**, knockdown of *PICALM* in HEK293 cells is associated with a significant increase in LDL receptor expression at the cell surface. This increase in surface LDL receptor protein correlates with the increase in LDL receptor mRNA transcript seen by qPCR (**Fig 2A and 2B**). Similar results were seen with a separate shRNA (shRNA15). Endocytosis assays showed an increase in the net amount of LDL receptor internalized in *PICALM* knockdown cells (**Fig 4B**). The efficiency of LDL receptor internalization, measured as the ratio of LDL receptor internalized to the amount initially present at the cell surface, was not altered in *PICALM* knockdown cells (**Fig 4C**).

## Discussion

Cholesterol is essential for cell survival. The majority of cellular cholesterol is found within the plasma membrane, where it regulates membrane rigidity and permeability, and modulates specific aspects of cell signaling [42–47]. Cholesterol homeostasis ensures a balance between internalization of extracellular cholesterol and endogenous synthesis. Cells scavenge cholesterol when complexed with lipoproteins. This internalization occurs predominantly through clathrin-mediated endocytosis (CME) of LDL, via a variety of receptors belonging to the LDL receptor family [41–43]. Once cholesterol is internalized, it is trafficked through the endosomal-lysosomal system, and released from the lysosome to the cytoplasm and various cellular compartments within the cell. If cholesterol internalization is blocked, or if there are defects in cholesterol trafficking, SREBP2, the main regulator of cholesterol biosynthesis gene expression, can activate expression of cholesterol biosynthesis genes to restore cellular cholesterol levels [41–43]. Importantly, maintaining cellular cholesterol homeostasis is essential, since defects can lead to a wide range of diseases such as cancer, atherosclerosis, and neurodegenerative disease [41, 48, 49].

We performed a microarray analysis of *Picalm*-deficient and *PICALM*-rescued murine hematopoietic cells and showed that PICALM perturbation appears to modulate cellular cholesterol biosynthesis gene expression. However, in this overexpression model, *PICALM* levels in rescued cells may have been supraphysiologic. Cells that express “wild type” levels of PICALM exhibit baseline levels of cholesterol biosynthesis genes. It is straightforward to understand how reduced PICALM levels could perturb expression by impairing normal PICALM activity. Previous studies have shown that PICALM overexpression can result in PICALM having a dominant negative effect on multiple cellular processes [3, 5]. Thus, both PICALM overexpression and PICALM reduction can result in a similar phenotype. To show that elevated PICALM expression levels alters cellular cholesterol homeostasis, we overexpressed PICALM in HEK293 cells. This resulted in elevated cholesterol metabolism gene expression, indicating that PICALM overexpression may exert a dominant negative effect on cholesterol homeostasis. To demonstrate that physiological levels of PICALM regulate cholesterol homeostasis, we knocked down PICALM expression using shRNAs. The results of **Fig 2** show that PICALM deficiency leads to elevated expression of cholesterol biosynthesis genes.

Multiple studies indicate that proteins involved in CME and endosomal-lysosomal trafficking can play key roles in maintaining normal cellular cholesterol homeostasis [50–55]. Given PICALM’s well-established contribution to CME and its possible role in cellular trafficking, [2, 14, 56] we explored how PICALM perturbation might modulate cellular cholesterol metabolism. To this end, we used ^13^C tracer analysis [39]. Our studies demonstrate that *PICALM* knockdown results in both elevated net cholesterol scavenging and increased total cellular cholesterol levels (**Fig 3**). Furthermore, several species of fatty acids also showed increased cellular pool sizes and increased net scavenging rates in the absence of PICALM (**S3 and S4 Figs**), indicating that PICALM may play a broader role in cellular lipid homeostasis. Interestingly, loss of PICALM did not result in a significant change in net cholesterol synthetic rate. This result could be explained by a variety of factors. It is known that increased cellular cholesterol can inhibit flux through the mevalonate cholesterol biosynthetic pathway via posttranscriptional mechanisms, such as degradation of HMG-CoA reductase [57] and squalene monooxygenase [58]. Alternatively, it is also possible that increased cholesterol levels result in heightened cholesterol efflux [59] in PICALM-deficient cells. In this scenario, heightened cholesterol efflux could result in an underestimation of the magnitude of change in gross synthetic rate between PICALM-sufficient and-deficient cells. Nevertheless, the concomitant increase of net cholesterol import, with activation of cholesterol biosynthesis pathway gene expression, suggests that cholesterol homeostasis is profoundly perturbed in *PICALM*-deficient cells [60, 61].

Our studies further demonstrate that reduced PICALM results in elevated LDL receptor surface expression and internalization (**Fig 4**). However, PICALM reduction does not appear to affect the efficiency of LDL receptor internalization (**Fig 4**). These results suggest that PICALM does not play an essential role in the internalization of the LDL receptor from the cell surface, but rather that PICALM may be required for the proper intracellular trafficking of internalized cholesterol. We propose that the elevated levels of LDL receptor surface expression and internalization are a result of increased expression of the LDL receptor, caused by perturbation of cholesterol homeostasis.

Several reports indicate that PICALM affects cellular trafficking through the endosomal-lysosomal system [13, 14, 56]. PICALM modulates the localization and activity of SNARE proteins such as VAMP3 and VAMP8, both of which are important for endosomal-lysosomal function and macroautophagy [62, 63]. Our previous studies have shown that PICALM’s ability to modulate the localization of VAMP3 and VAMP8, results in perturbed macroautophagy. [13]. Intriguingly, these SNARE proteins have been shown to be mislocalized in Niemann-Pick Type C (NPC) disease, which is characterized by perturbed endosomal-lyososomal trafficking of cholesterol and altered cholesterol homeostasis [60, 64–66]. It has also been demonstrated that macroautophagy is altered in NPC disease [63–65]. Collectively, this suggests that an alteration in PICALM levels can lead to altered cellular cholesterol homeostasis by mislocalization of VAMP3 and VAMP8 SNARE proteins, which contribute to both improper cholesterol trafficking and macroautophagy [64, 67]. Perturbation of both macroautophagy and cholesterol trafficking may contribute to the elevated cholesterol biosynthesis gene expression, LDL receptor surface expression and internalization, and cholesterol uptake, as the cells are unable to sense cellular cholesterol in the absence of PICALM. Deciphering the precise mechanism is beyond the scope of this study.

Our observations suggest that PICALM plays a regulatory role in maintaining cellular cholesterol homeostasis. In addition, they support a novel mechanism by which PICALM might contribute to disease. Multiple independent Genome Wide Association Studies (GWAS) indicate that PICALM SNPs play a role in the development of late-onset Alzheimer’s Disease (AD) [21–24]. However, how these SNPs affect PICALM expression levels or function is poorly understood; some studies suggest that PICALM is overexpressed, while others indicate that a reduction in PICALM activity can play a pathogenic role in AD [68–71]. Alterations in PICALM levels may modulate amyloid beta protein formation and Tau accumulation [13, 25]. The present work suggests that the ability of PICALM to modulate cholesterol biology might be an additional mechanism by which PICALM contributes to AD. This is consistent with a number of studies that have linked perturbed cholesterol metabolism to late-onset AD, as well as other neurodegenerative diseases [72–79].

The expression of APOE, a key lipoprotein and carrier of cholesterol in the brain, has been shown to be a highly significant risk factor in the development of late-onset AD [23, 80]. Meta-analysis studies suggest that PICALM and APOEε4 may synergistically interact to help promote the development of AD [22, 81]. However, the manner by which APOE and PICALM might act together to promote the development of AD are not understood. Our work suggests that these risk factors may cooperate to promote the development of AD through the modulation of cellular cholesterol metabolism.

In summary, the studies presented here provide evidence to suggest that PICALM modulates cellular cholesterol metabolism. Loss of PICALM activates cellular cholesterol biosynthesis gene expression, increases LDL receptor surface expression and internalization, enhances cholesterol uptake, and increases total cellular cholesterol. This phenotype suggests that cholesterol sensing is abnormal when PICALM activity is perturbed. This novel role of PICALM in cellular cholesterol homeostasis has implications in understanding how PICALM might contribute to disease.

## Supporting Information

S1 Fig shRNA knockdown of *PICALM* does not lead to altered cholesterol biosynthesis gene expression, when compared to control cells, in lipid deficient conditions. Cholesterol biosynthesis gene expression was measured by qPCR in cells expressing a control (*luciferase*) shRNA or *PICALM* shRNAs (shRNA4). Analysis was performed in HEK293 cells grown in normal conditions (10% serum–fetal bovine serum, FBS) (n = 6, n = 5 for Hmgcr) or 10% lipid deficient serum (LPDS) for 5 hours (n = 3) or 24 hours (n = 3). Expression levels were normalized to those of shRNA control cells in normal conditions. *p<0.05
(TIFF)

S2 Fig shRNA knockdown of *PICALM* results in modest elevation of the mature form of SREBP2. **(A)** The precursor (120 kDa) and SREBP2 mature form (70 kDa) were measured by immunoblot in HEK293 cells expressing shRNA15 or a control shRNA. A higher exposure of the precursor form is shown to facilitate its visualization. Triplicate cell lysates were analyzed in 3 separate experiments. Quantitation of SREBP2 precursor and mature forms were normalized to actin, with values shown relative to levels in shRNA control cells. The uncropped western blot is provided in **S5 Fig. (B)** The precursor (120 kDa) and mature form of SREBP2 (70 kDa) were measured by immunoblot in HEK293 cells grown in normal, 10% serum (fetal bovine serum, FBS) or 10% Lipid deficient serum (LPDS) for 24 hours, n = 3 *p<0.05.
(TIFF)

S3 Fig Total Fatty acid levels are maintained or elevated in the absence of PICALM. Total fatty acid levels were measured in HEK293 cells expressing a *luciferase* shRNA (shRNALuc) control or *PICALM* shRNA (shRNA4), in media containing a 1:1 molar ratio of natural to U-^13^C_6_-glucose. Fatty acid abbreviations are indicated in **S3 Table**. All results were normalized to cell number. n = 4 in each experimental group *p<0.05.
(TIFF)

S4 Fig Net fatty acid scavenging, synthesis, and total accumulation are maintained or elevated in the absence of PICALM. Net rates of fatty acid synthesis, scavenging and accumulation were calculated for shRNA luciferase (shRNALuc) control and *PICALM* knockdown (shRNA4) HEK293 cells in media containing a 1:1 molar ratio of natural to U-^13^C_6_-glucose using cellular pool size, relative contribution of synthesis and scavenging, and doubling time of cells as described in materials and methods. n = 4 in each experimental group; *p<0.05.
(TIFF)

S5 Fig Uncropped Western Blots. All western blots within the paper have been provided in uncropped form. Boxes around bands indicate the lanes of the western blot which were shown within the manuscript. Western blots were shown at high exposure in order for the molecular size markers to be visible.
(TIFF)

S1 Table Sequences of Murine Primers for qPCR (TIFF)

S2 Table Sequences of Human Primers for qPCR (TIFF)

S3 Table List of Analytes and Internal Standards for GC/MS (TIFF)

S4 Table Top 40 Up- regulated Genes (PDF)

S5 Table Top 40 Down-regulated Genes (PDF)
