# Supplementary figures and images for: Modulation of PICALM Levels Perturbs Cellular Cholesterol Homeostasis
Source: PLoS One. 2015 Jun 15;10(6):e0129776. doi: 10.1371/journal.pone.0129776 (PMC4467867; doi:10.1371/journal.pone.0129776)

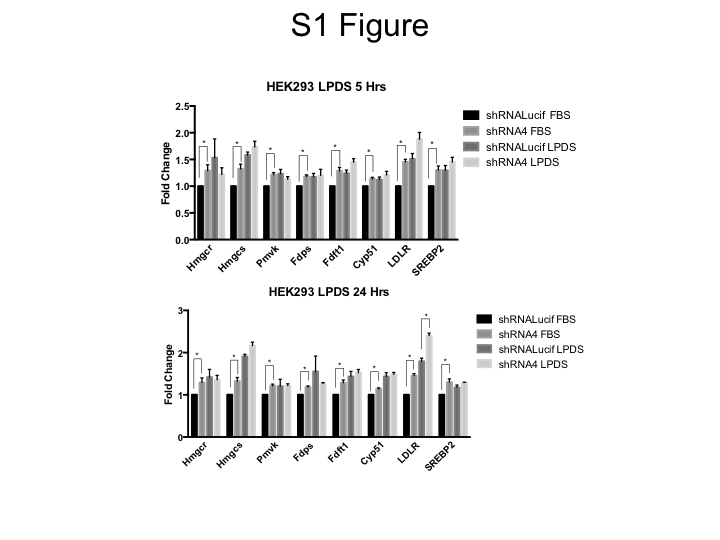

Supplement: S1 Fig — Cholesterol biosynthesis gene expression was measured by qPCR in cells expressing a control (luciferase) shRNA or PICALM shRNAs (shRNA4). Analysis was performed in HEK293 cells grown in normal conditions (10% serum–fetal bovine serum, FBS) (n = 6, n = 5 for Hmgcr) or 10% lipid deficient serum (LPDS) for 5 hours (n = 3) or 24 hours (n = 3). Expression levels were normalized to those of shRNA control cells in normal conditions. *p<0.05 (TIFF) [file pone.0129776.s001.tiff]

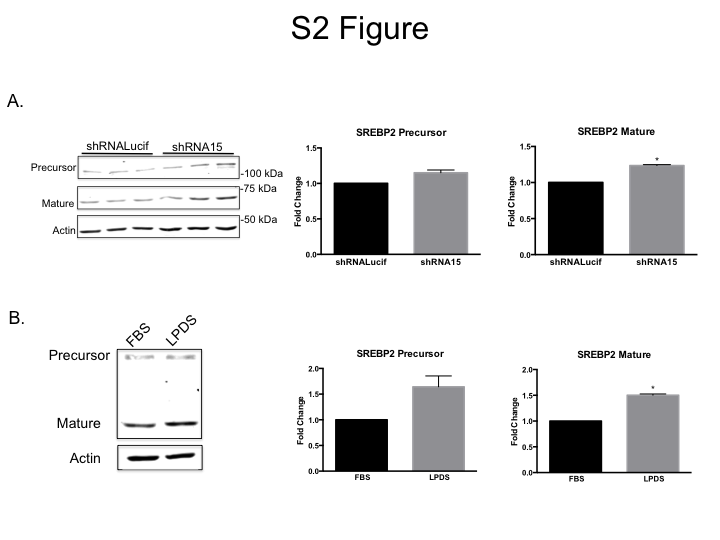

Supplement: S2 Fig — (A) The precursor (120 kDa) and SREBP2 mature form (70 kDa) were measured by immunoblot in HEK293 cells expressing shRNA15 or a control shRNA. A higher exposure of the precursor form is shown to facilitate its visualization. Triplicate cell lysates were analyzed in 3 separate experiments. Quantitation of SREBP2 precursor and mature forms were normalized to actin, with values shown relative to levels in shRNA control cells. The uncropped western blot is provided in S5 Fig. (B) The precursor (120 kDa) and mature form of SREBP2 (70 kDa) were measured by immunoblot in HEK293 cells grown in normal, 10% serum (fetal bovine serum, FBS) or 10% Lipid deficient serum (LPDS) for 24 hours, n = 3 *p<0.05. (TIFF) [file pone.0129776.s002.tiff]

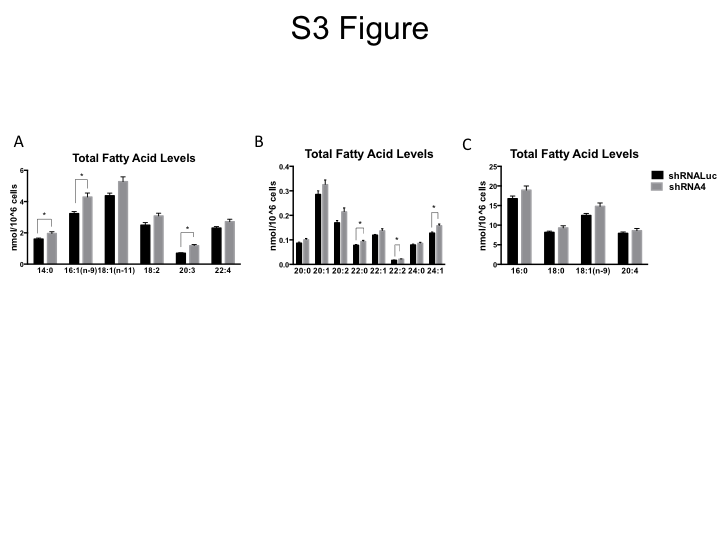

Supplement: S3 Fig — Total fatty acid levels were measured in HEK293 cells expressing a luciferase shRNA (shRNALuc) control or PICALM shRNA (shRNA4), in media containing a 1:1 molar ratio of natural to U-13C6-glucose. Fatty acid abbreviations are indicated in S3 Table. All results were normalized to cell number. n = 4 in each experimental group *p<0.05. (TIFF) [file pone.0129776.s003.tiff]

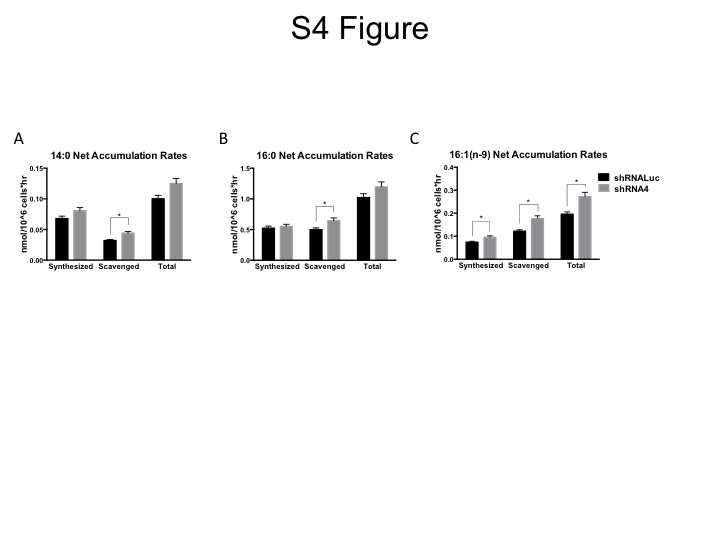

Supplement: S4 Fig — Net rates of fatty acid synthesis, scavenging and accumulation were calculated for shRNA luciferase (shRNALuc) control and PICALM knockdown (shRNA4) HEK293 cells in media containing a 1:1 molar ratio of natural to U-13C6-glucose using cellular pool size, relative contribution of synthesis and scavenging, and doubling time of cells as described in materials and methods. n = 4 in each experimental group; *p<0.05. (TIFF) [file pone.0129776.s004.tiff]

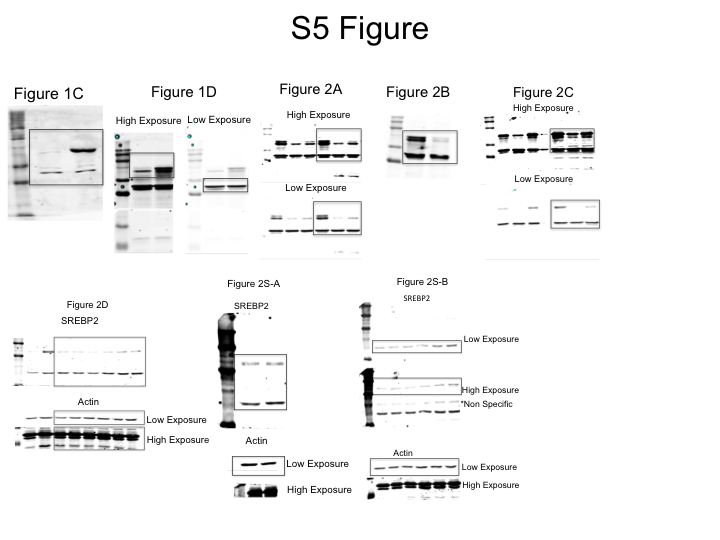

Supplement: S5 Fig — All western blots within the paper have been provided in uncropped form. Boxes around bands indicate the lanes of the western blot which were shown within the manuscript. Western blots were shown at high exposure in order for the molecular size markers to be visible. (TIFF) [file pone.0129776.s005.tiff]

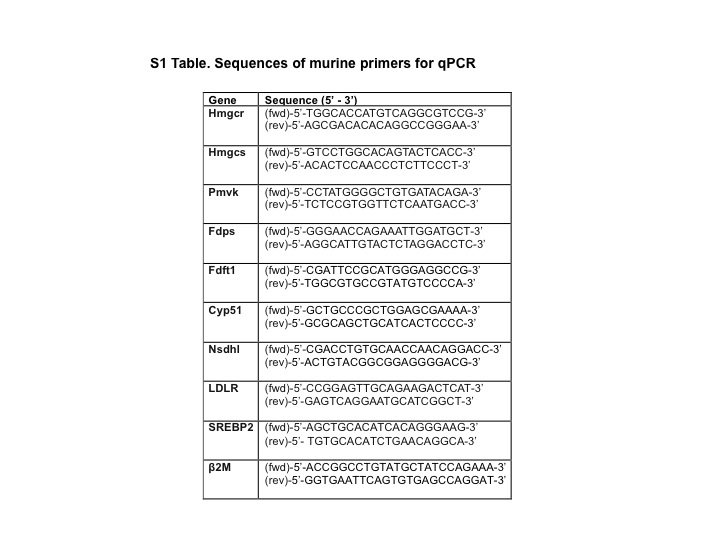

Supplement: S1 Table — (TIFF) [file pone.0129776.s006.tiff]

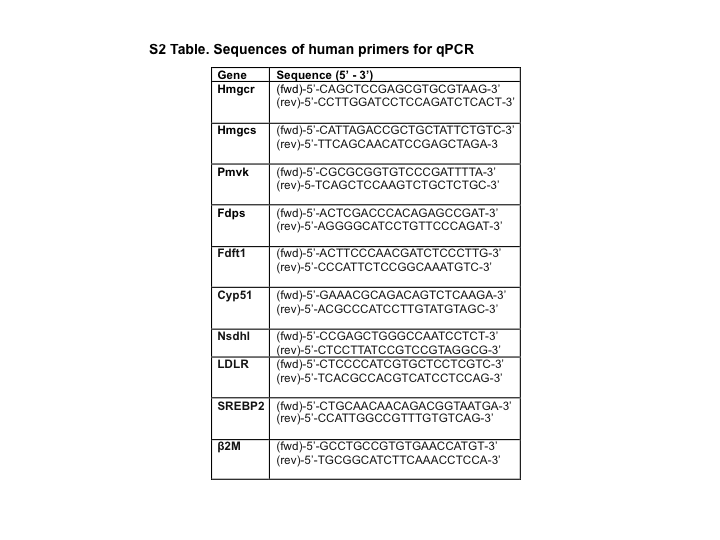

Supplement: S2 Table — (TIFF) [file pone.0129776.s007.tiff]

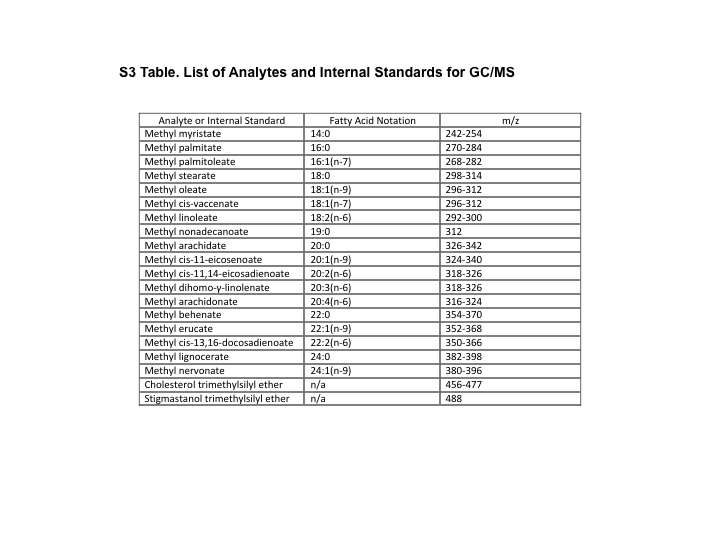

Supplement: S3 Table — (TIFF) [file pone.0129776.s008.tiff]
