# Supplementary material for: Modulation of PICALM Levels Perturbs Cellular Cholesterol Homeostasis
Source: PLoS One. 2015 Jun 15;10(6):e0129776. doi: 10.1371/journal.pone.0129776 (PMC4467867; doi:10.1371/journal.pone.0129776)
Supplement: S4 Table — (PDF) [file pone.0129776.s009.pdf]

S4 Supplementary Table. Top 40 Genes Upregulated in Microarray

Rank-Highest Fold Change	Gene ID	Gene Name	Fold Change
1	1419691_at	Camp	5.020108341
2	1419709_at	Stfa1 /// Stfa3	4.305725956
3	1418722_at	Ngp	4.017798606
4	1427747_a_at	Lcn2	3.563405709
5	1434484_at	1100001G20Rik	3.336776368
6	1450009_at	Ltf	2.905153714
7	1448756_at	S100a9	2.88053193
8	1449829_at	Itgb2l	2.707390051
9	1417290_at	Lrg1	2.686855994
10	1427256_at	Vcan	2.602486627
11	1455099_at	Mogat2	2.370766785
12	1455493_at	Syne1	2.366002339
13	1423915_at	Olfml2b	2.302044944
14	1418345_at	Tnfsf12 /// Tnfsf12-tnfsf13 /// Tnfsf13	2.281527827
15	1421811_at	LOC640441 /// Thbs1	2.205576804
16	1422013_at	Clec4a2	2.168780806
17	1426642_at	Fn1	2.140681632
18	1451858_at	LOC668727 /// Mrgpra2	2.134156538
19	1422756_at	Slc32a1	2.119415944
20	1460302_at	Thbs1	2.118788378
21	1439036_a_at	Atp1b1	2.104710053
22	1420407_at	Ltb4r1	2.072162652
23	1425538_x_at	Ceacam1	2.042613152
24	1449038_at	Foxo3	1.952038152
25	1460419_a_at	Prkcb1	1.932432471
26	1449184_at	Pglyrp1	1.925134509
27	1425407_s_at	Clec4a2 /// Clec4b1	1.923493894
28	1460682_s_at	Ceacam1 /// Ceacam2	1.90729632
29	1456603_at	1500005K14Rik	1.711995904
30	1450389_s_at	Pip5k1b	1.670486704
31	1434674_at	Lyst	1.656431737
32	1422046_at	Itgam	1.650341972
33	1451021_a_at	Klf5	1.631513019
34	1420641_a_at	Sqrdl	1.620140017
35	1448871_at	Mapk13	1.617675514
36	1434025_at	---	1.605362664
37	1422678_at	Dgat2	1.603516835
38	1425958_at	Il1f9	1.597546584
39	1450377_at	LOC640441 /// Thbs1	1.597261194
40	1449453_at	Bst1	1.565534444
