# Supplementary material for: Modulation of PICALM Levels Perturbs Cellular Cholesterol Homeostasis
Source: PLoS One. 2015 Jun 15;10(6):e0129776. doi: 10.1371/journal.pone.0129776 (PMC4467867; doi:10.1371/journal.pone.0129776)
Supplement: S5 Table — (PDF) [file pone.0129776.s010.pdf]

S5 Supplementary Table. Top 40 Genes Downregulated in Microarray

Rank-Highest Fold Change	Gene ID	Gene Name	Fold Change
1	1437279_x_at	Sdc1	0.290652908
2	1436759_x_at	Cnn3 /// LOC100047856	0.353851269
3	1416645_a_at	Afp	0.41948489
4	1449939_s_at	Dlk1	0.427678845
5	1425923_at	Mycn	0.461652675
6	1425470_at	---	0.471932113
7	1448734_at	Cp	0.52079175
8	1437025_at	Cd28 /// LOC100048845	0.55841709
9	1425742_a_at	Tsc22d1	0.573457311
10	1419561_at	Ccl3	0.5834291
11	1454971_x_at	Tsc22d1	0.593925035
12	1421492_at	Ptgds2	0.600920586
13	1424733_at	P2ry14	0.605989835
14	1425454_a_at	Il12a	0.616812967
15	1434500_at	Ttyh2	0.62069503
16	1434149_at	Tcf4	0.63010886
17	1419387_s_at	Muc13	0.632879503
18	1418872_at	Abcb1b	0.635388992
19	1421027_a_at	Mef2c	0.643451471
20	1436917_s_at	Gpsm1	0.660352155
21	1420505_a_at	Stxbp1	0.6698788
22	1438659_x_at	Chchd6	0.682294149
23	1420824_at	Sema4d	0.69909734
24	1455090_at	Angptl2	0.699801906
25	1426543_x_at	Endod1	0.709220553
26	1417331_a_at	Arl6	0.730217171
27	1450082_s_at	Etv5	0.7355738
28	1426169_a_at	Lat2	0.73899666
29	1452679_at	Tubb2b	0.739272678
30	1433655_at	Rnf141	0.747678913
31	1454699_at	LOC100047324 /// Sesn1	0.75673627
32	1424029_at	Tspyl4	0.758446593
33	1451063_at	Stxbp4	0.759805468
34	1424242_at	Bphl	0.760188971
35	1455653_at	Ccnj	0.763337779
36	1438354_x_at	Cnn3	0.767225193
37	1426934_at	Nhsl1	0.771088042
38	1460695_a_at	2010111I01Rik	0.772714834

39	1425934_a_at	B4galt4	0.780265491
40	1451474_a_at	Parp8	0.784824373
